# Supplementary material for: Superelasticity of a photo-actuating chiral salicylideneamine crystal
Source: Commun Chem. 2022 Jan 10;5:4. doi: 10.1038/s42004-021-00618-8 (PMC9814393; doi:10.1038/s42004-021-00618-8)
Supplement: Supplementary file 1 — Supplementary Information [file 42004_2021_618_MOESM1_ESM.pdf]

Supplementary Information

Superelasticity of a photo-actuating chiral salicylideneamine crystal

Takuya Taniguchi,*[1] Kazuki Ishizaki,[2] Daisuke Takagi,[3] Kazuki Nishimura,[4] Hiroki Shigemune,[4] Masahiro Kuramochi,[5] Yuji C. Sasaki,[6] Hideko Koshima,[7] Toru Asahi[2,3,7]

[1] Center for Data Science, Waseda University, 1-6-1 Nishiwaseda, Shinjuku-ku, Tokyo 169-0051, Japan

[2] Department of Advanced Science and Engineering, Graduate School of Advanced Science and Engineering, Waseda University, 3-4-1 Okubo, Shinjuku-ku, Tokyo 169-8555, Japan

[3] Department of Life Science and Medical Bioscience, Graduate School of Advanced Science and Engineering, Waseda University, 3-4-1 Okubo, Shinjuku-ku, Tokyo 169-8555, Japan

[4] Department of Electrical Engineering, Shibaura Institute of Technology, 3-7-5 Toyosu, Koto-ku, Tokyo, 135-8548 Japan

[5] Department of Materials Science and Engineering, Graduate School of Science and Engineering, Ibaraki University, 4-12-1 Naka-narusawa-cho, Hitachi-shi, Ibaraki, 316-8511 Japan

[6] Graduate School of Frontier Sciences, The University of Tokyo, Kashiwa, 277-8561, Japan

[7] Research Organization for Nano & Life Innovation, Waseda University, 513 Wasedatsurumaki-cho, Shinjuku-ku, Tokyo 162-0041, Japan

* Correspondence to takuya.taniguchi@aoni.waseda.jp

Contents

1. **Elastic properties and actuation**

   Supplementary Note 1. Calculations of mechanical properties. Page S3

   Supplementary Note 2. Calculations of actuation performance. Page S4

   Figure S1. Repeatability of initial load applying on (001)/($00\bar{1}$) face. Page S5

   Figure S2. Measurement of Young's modulus loaded on side and cross-section faces. Page S6

   Figure S3. Cleavage of enol-(*S*)-**1** crystals by loading. Page S7

   Figure S4. Twist handedness of twisted bending. Page S8

2. **Condition of PtPT**

   Figure S5. Calculated and measured IR spectra. Page S9

   Figure S6. Dependence of light intensity on the blocking force. Page S10

   Figure S7. Surface temperature change due to the photothermal effect. Page S11

   Figure S8. Phase transition due to the photohtermal effect. Page S12

   Figure S9. Size effect on PtPT. Page S13

3. **Finite element analysis (FEA)**

   Figure S10. Settings and parameters for FEA model. Page S14

   Figure S11. Mechanism of different twisting handedness. Page S15

   Figure S12. Parameter optimization of FEA simulation when (001) face was irradiated. Page S16

   Figure S13. FEA simulation crystal deformation irradiated on ($00\bar{1}$) face. Page S17

   Figure S14. Parameter optimization of FEA simulation when ($00\bar{1}$) face was irradiated. Page S18

   Figure S15. Comparison of experiment and simulation of other crystals. Page S19

4. **Polarized microscopy**

   Figure S16. Phase boundary at PtPT and reverse transition. Page S20

   Figure S17. Anisotropy of PtPT propagation. Page S21

5. **Diffracted X-ray blinking (DXB)**

   Figure S18. Outline of DXB measurement. Page S22

   Supplementary Note 3. Explanation of DXB. Page S23

1. Elastic properties and actuation

Supplementary Note 1. Calculations of mechanical properties.

As to mechanical properties, when a free beam fixed with an edge is loaded P at the distance l from the edge, the beam responds by bending with the displacement δ. From the response in elastic linear region, Young's modulus E can be calculated by following equation:

$$E = \frac{Pl^3}{3\delta I} \tag{1.1}$$

here, I is the moment of inertia of rectangle cross-section as following

$$I = \frac{bh^3}{12} \tag{1.2}$$

with width b and thickness h. Also, section modulus Z is

$$Z = \frac{bh^2}{6} \tag{1.3}$$

and the bending moment M is dependent on the distance from the loaded position, and the maximum at fixed position is

$$M = Pl. \tag{1.4}$$

From these equations, the maximum stress σ is expressed as

$$\sigma = \frac{M}{Z} = \frac{6Pl}{bh^2} \tag{1.5}$$

and the strain ε is

$$\varepsilon = \frac{6Pl}{bh^2 E} \tag{1.6}$$

When the cross-section area is loaded by compression mode, the strain, stress, and Young's modulus are expressed as following

$$E = \frac{\sigma}{\varepsilon} \tag{1.7}$$

$$\varepsilon = \frac{\Delta l}{l} \tag{1.8}$$

$$\sigma = \frac{P}{bh}. \tag{1.9}$$

Supplementary Note 2. Calculations of actuation performance.

Actuation performance of an enol-(*S*)-**1** crystal was evaluated based on the region of the simple bending after completing PtPT, because torsional bending was difficult to be incorporated into the calculations. Maximum displacement was obtained without loading, and maximum force was obtained by blocking the bending. Strain ε and stress σ can be calculated using crystal size, Young's modulus E, and maximum displacement of simple bending. Then, elastic energy density W (J m^{-3})

$$W = \frac{1}{2}\sigma\varepsilon \tag{2.1}$$

was calculated. Power density P (W m^{-3}) was calculated based on the time to reach maximum displacement,

$$P = \frac{W}{t} \tag{2.2}$$

Energy efficiency η was calculated as the ratio of output energy over input energy,

$$\eta = \frac{P_{out}}{P_{in}} = \frac{WV}{LAt} \tag{2.3}$$

where V is crystal volume (m^3), L is light intensity (W m^{-2}), and A is irradiated area (m^2).

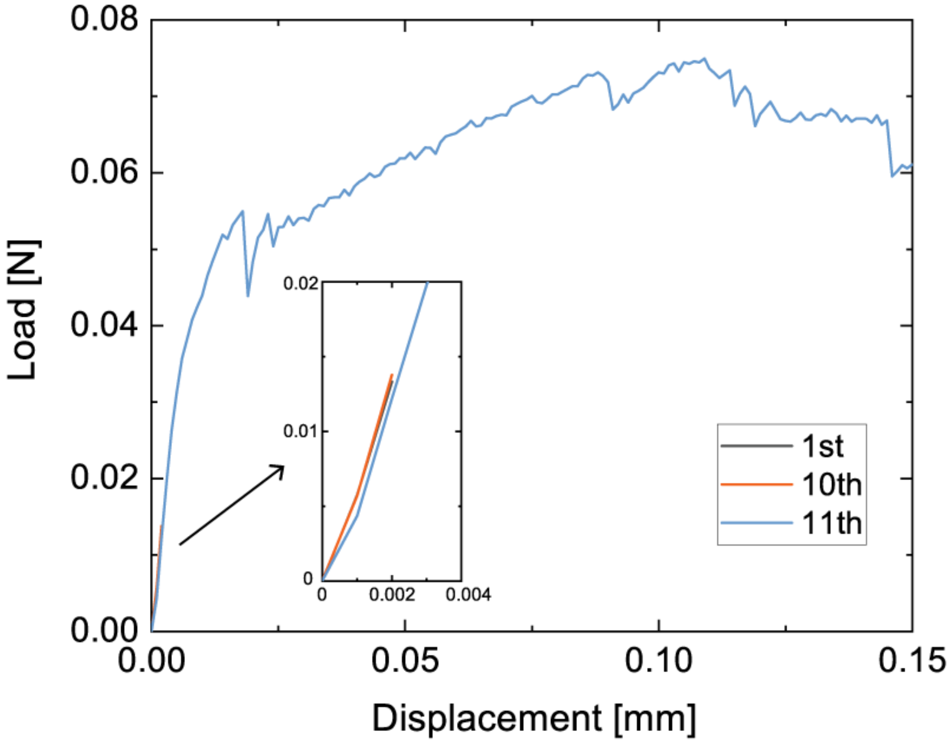

Supplementary Figure 1. Repeatability of initial load applying on (001)/($00\bar{1}$) face. Small load up to 13 mN was repeatedly applied 10 times as shown in the inset figure, and then load was continuously applied at 11th trial.

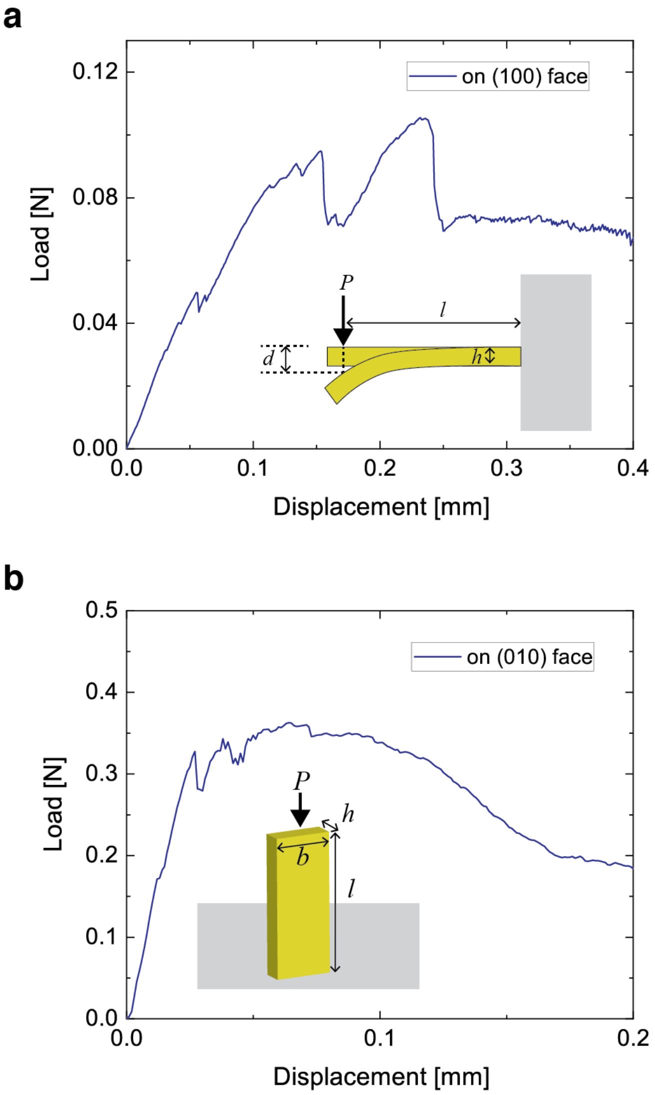

Supplementary Figure 2. **Measurement of Young's modulus loaded on side and cross-section faces**. **a,b** Typical load-displacement curves of enol-(*S*)-**1** crystals loaded on (100)/($\bar{1}$00) side face measured by bending (**a**), and (010)/(0$\bar{1}$0) cross section face measured by compression (**b**).

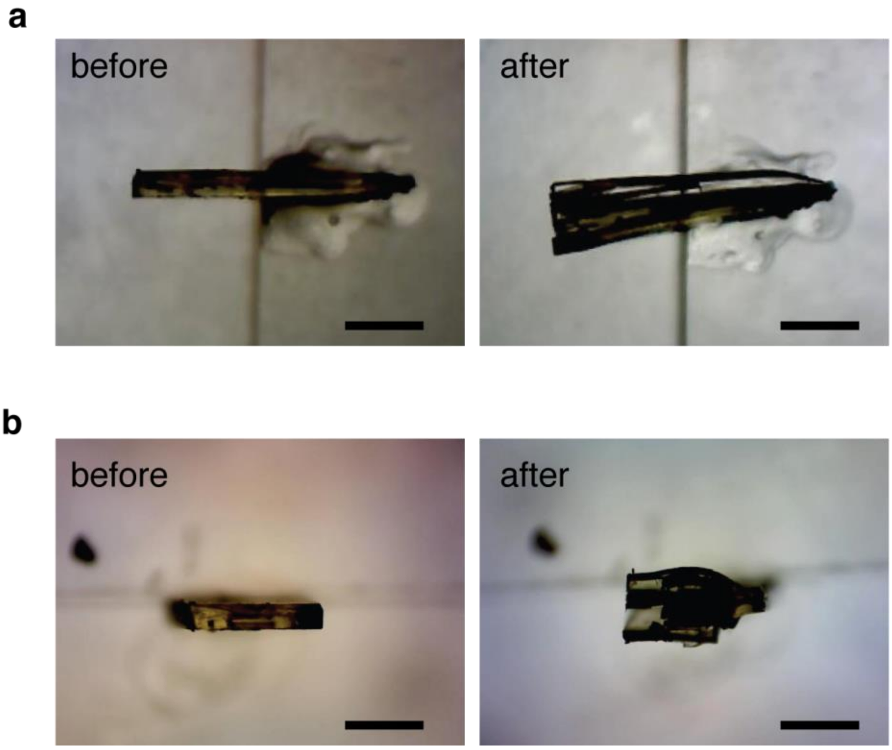

Supplementary Figure 3. Cleavage of enol-(*S*)-1 crystals by loading. a Photographs before and after loading on (100)/($\bar{1}$00) side face. **b** Photographs before and after loading on (010)/(0$\bar{1}$0) cross-section face. Scale bars are 1 cm.

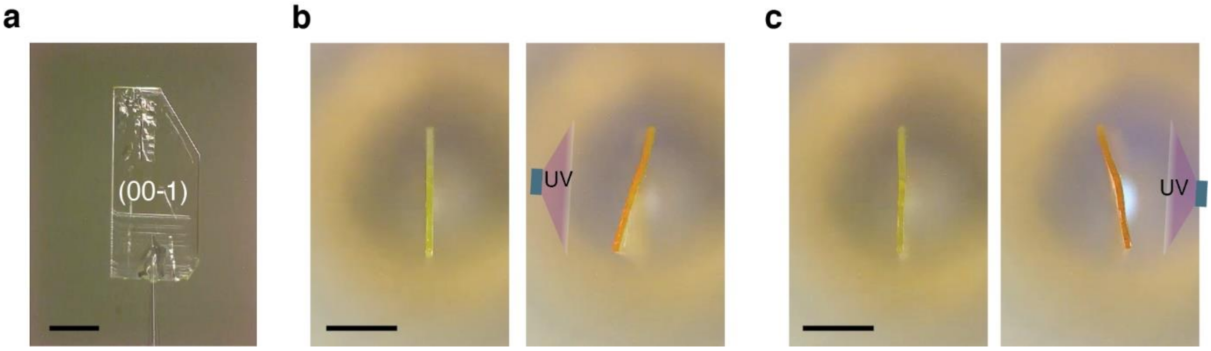

Supplementary Figure 4. Twist handedness of twisted bending. **a** Top (00$\bar{1}$) face view of an enol-(*S*)-**1** crystal (3.8 mm, 1.8 mm, 96 μm). **b** Twisted bending with left-handed twist of the crystal when (001) face was irradiated by UV light. **c** Twisted bending with right-handed twist of the crystal when (00$\bar{1}$) face was irradiated by UV light. Scale bars are 1 mm.

2. Condition of PtPT

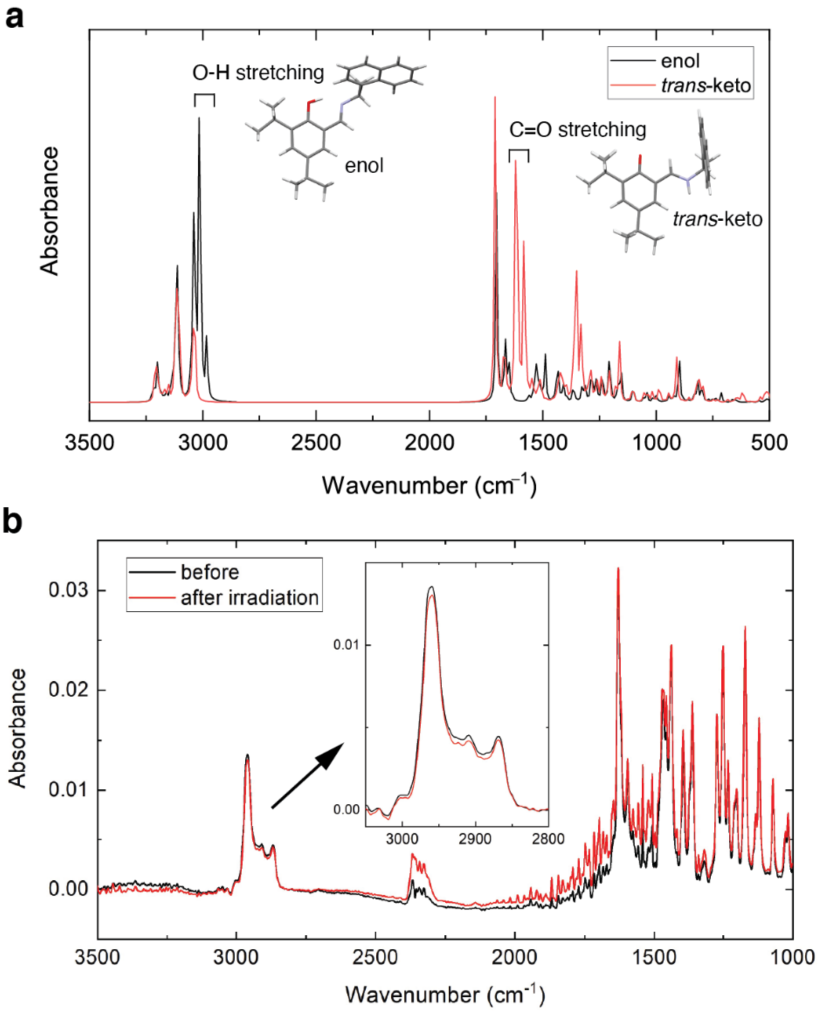

Supplementary Figure 5. Calculated and measured IR spectra. a Calculated IR spectra of enol and *trans*-keto form, computed at the level of B3LYP/6-31G(d,p) using Gaussian 09. **b** IR spectra before and under UV light of 180 mW cm^{-2}.

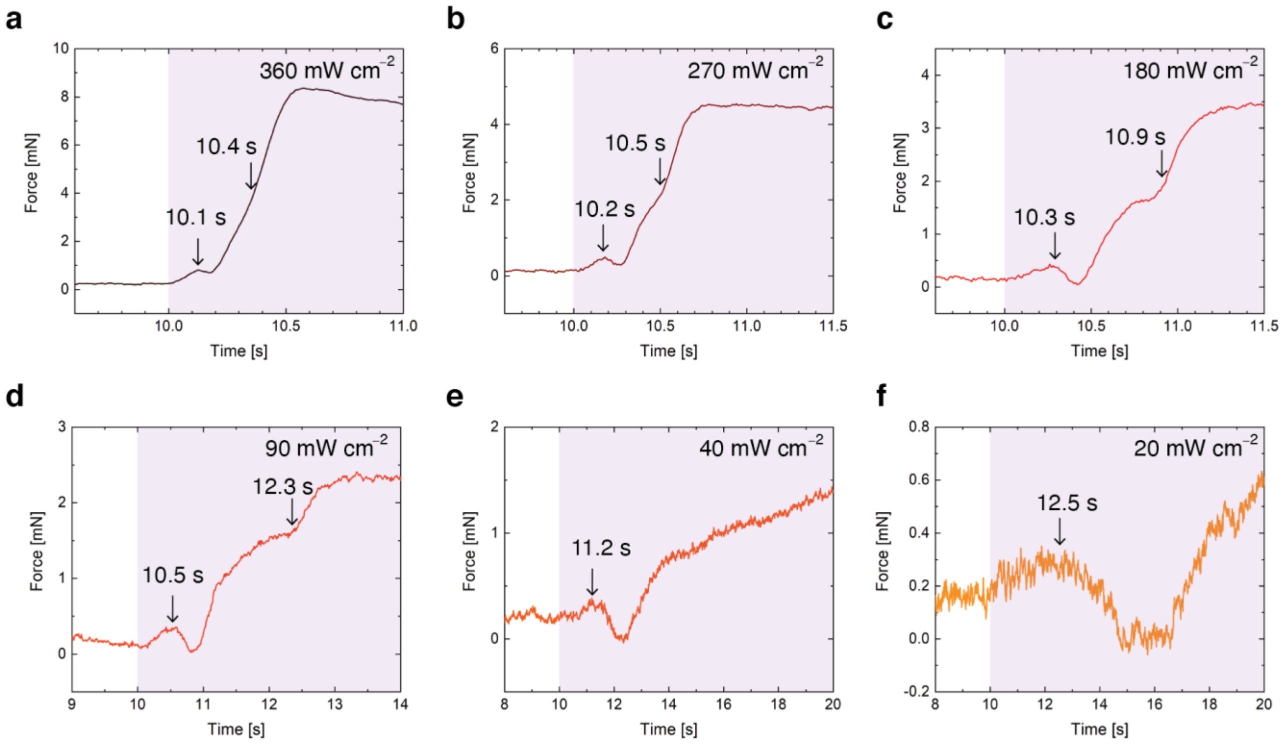

Supplementary Figure 6. Dependence of light intensity on the blocking force. a–f Bolcking force of the enol-(*S*)-**1** crystal, used in Figure 4, when ($00\bar{1}$) face was irradiated by UV light of different intensities. The first peak should indicate the beginning of PtPT, and the slight flat area should indicate the end of PtPT. The start and end of PtPT was faster as the light intensity was stronger. When weaker intensities of 20 and 40 mW cm^{-2}, the flat area did not appear, and in fact, torsional shape remained until the end of photo-irradiation in recorded movies.

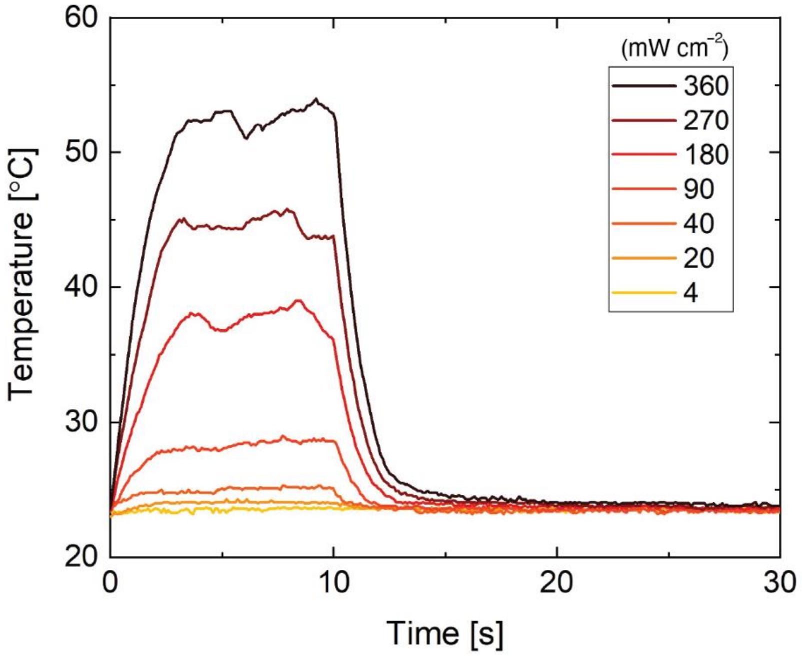

Supplementary Figure 7. Surface temperature change due to the photothermal effect. The surface temperature of the crystal, shown in Figure 4 in the main text, was measured by IR thermography. UV light irradiation was conducted in 0–10 s.

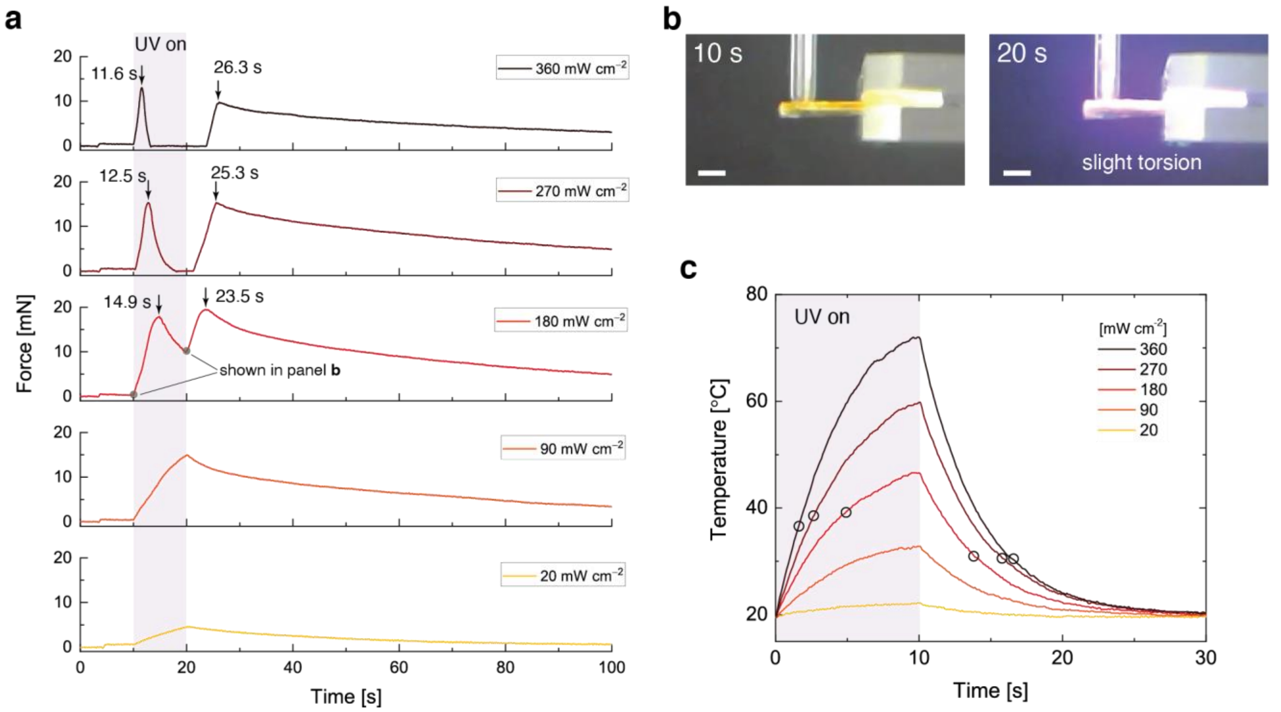

Supplementary Figure 8. Phase transition due to the photohtermal effect. a Time dependence of force generation of a larger crystal of enol-(*S*)-**1** (3.3 × 3.3 × 0.27 mm^3) upon UV light irradiation of various intensities. **b** Photographs of the crystal before and under light irradiation of 180 mW cm^{-2}. Scale bars are 1 mm. Slight torison remains at 20 s due to the imcopletion of phase transition. **c** Surface temperature increaese of the crystal upon light irradiation. Circles represent the timing shown by arrows in panel **a**. This relaitonship of force and temperature suggests that the phase transition was induced most probably by the photothermal effect.

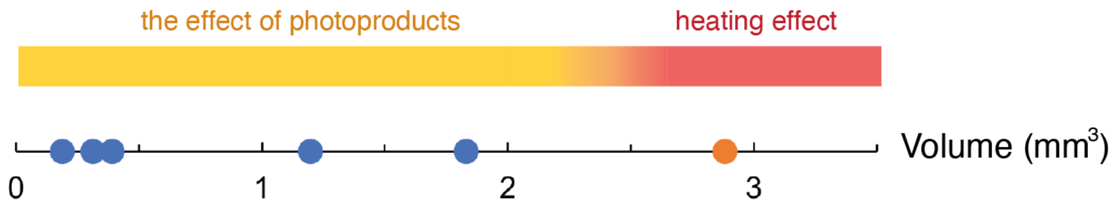

Supplementary Figure 9. **Size effect on PtPT**. Smaller crystals exhibit the phase transition by the effect of photoproducts (*i.e.,* PtPT), and large crystal exhibits phase transition by heating effect (*i.e.*, thermal transition). The effect of photoproducts or heating was confirmed by time-series behaviors of force and temperature increase.

3. Finite element analysis (FEA)

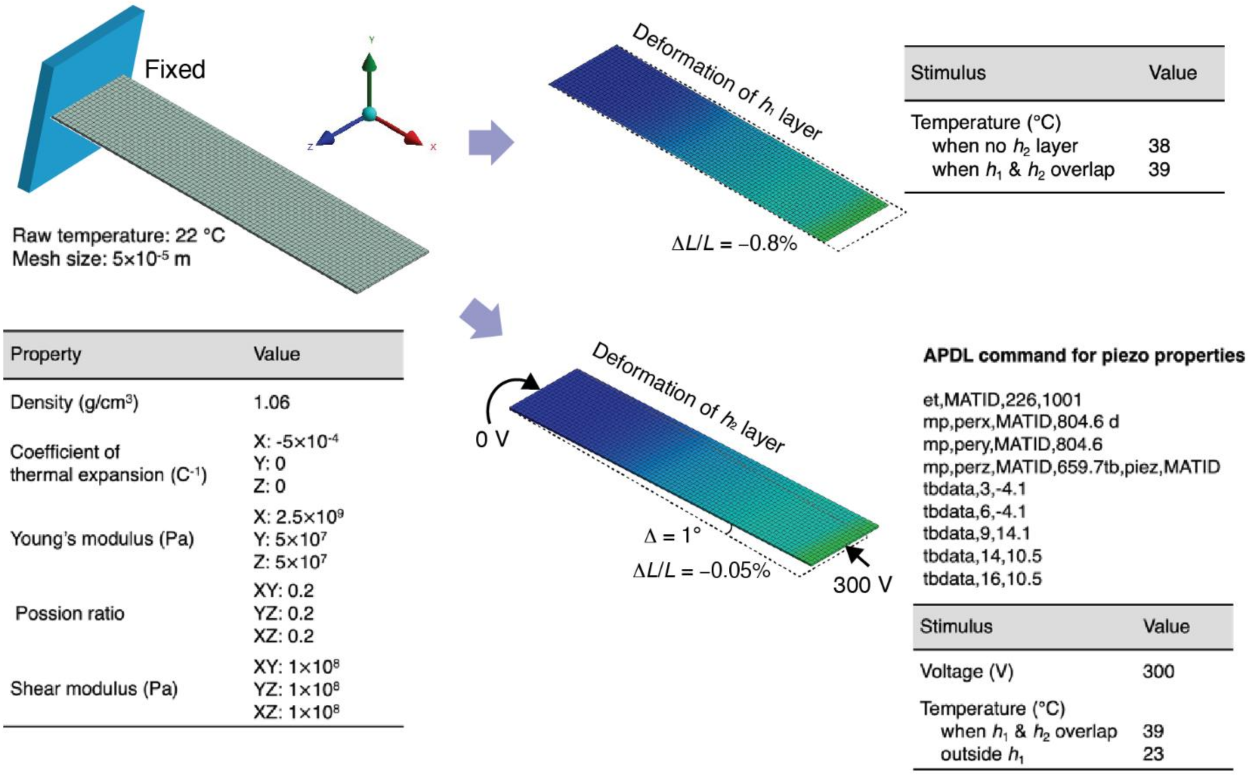

Property	Value
Density (g/cm^3)	1.06
Coefficient of thermal expansion (C^{-1})	X: -5×10^{-4}<br>Y: 0<br>Z: 0
Young's modulus (Pa)	X: 2.5×10^{9}<br>Y: 5×10^{7}<br>Z: 5×10^{7}
Possion ratio	XY: 0.2<br>YZ: 0.2<br>XZ: 0.2
Shear modulus (Pa)	XY: 1×10^{8}<br>YZ: 1×10^{8}<br>XZ: 1×10^{8}

Stimulus	Value
Temperature (°C)	
when no h_2 layer	38
when h_1 & h_2 overlap	39

Stimulus	Value
Voltage (V)	300
Temperature (°C)	
when h_1 & h_2 overlap	39
outside h_1	23

Supplementary Figure 10. Settings and parameters for FEA model. Material parameters are defined before FEA (left figure and table). Then, the deformation effects of h_1 and h_2 layers were defined for static analysis (right figures and tables). The effects of photoisomerization and PtPT were incorporated by negative thermal expansion and piezo effect, respectively.

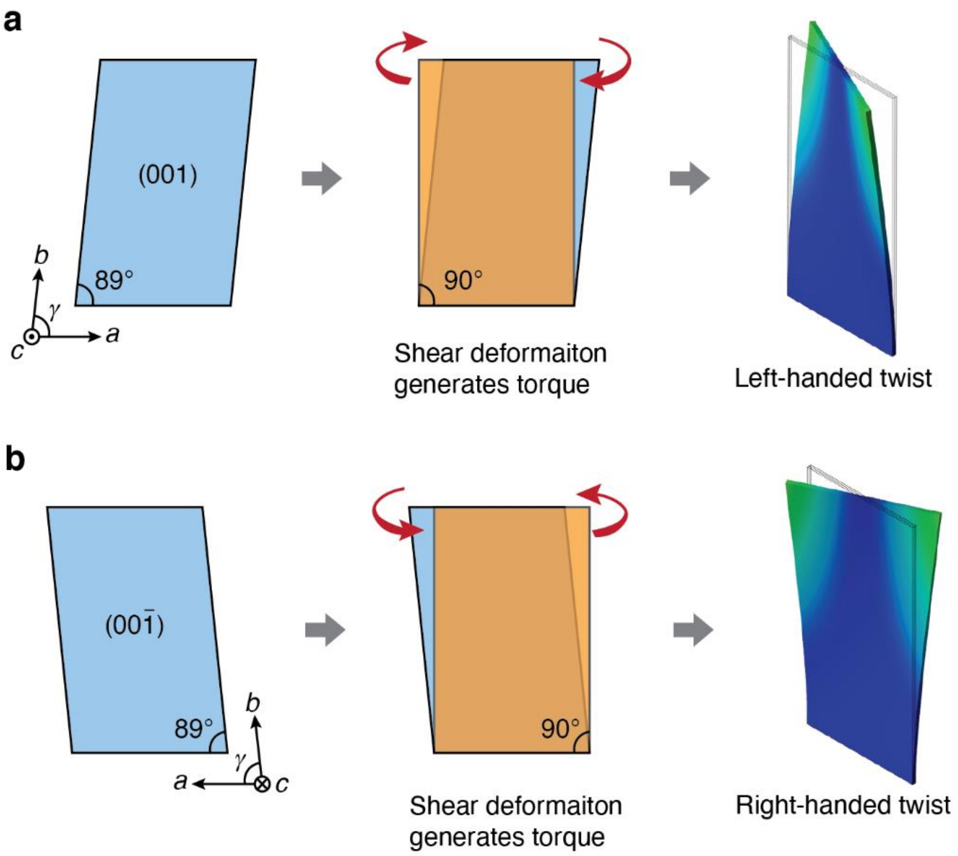

Supplementary Figure 11. Mechanism of different twisting handedness. **a** Left-handed twist due to shear deformed layer, which imitates PtPT when irradiated on (001) face. **b** Right-handed twist due to shear deformed layer, which imitates PtPT when irradiated on (00$\bar{1}$) face. In either case, length change was ignored for simply discussing twist handedness.

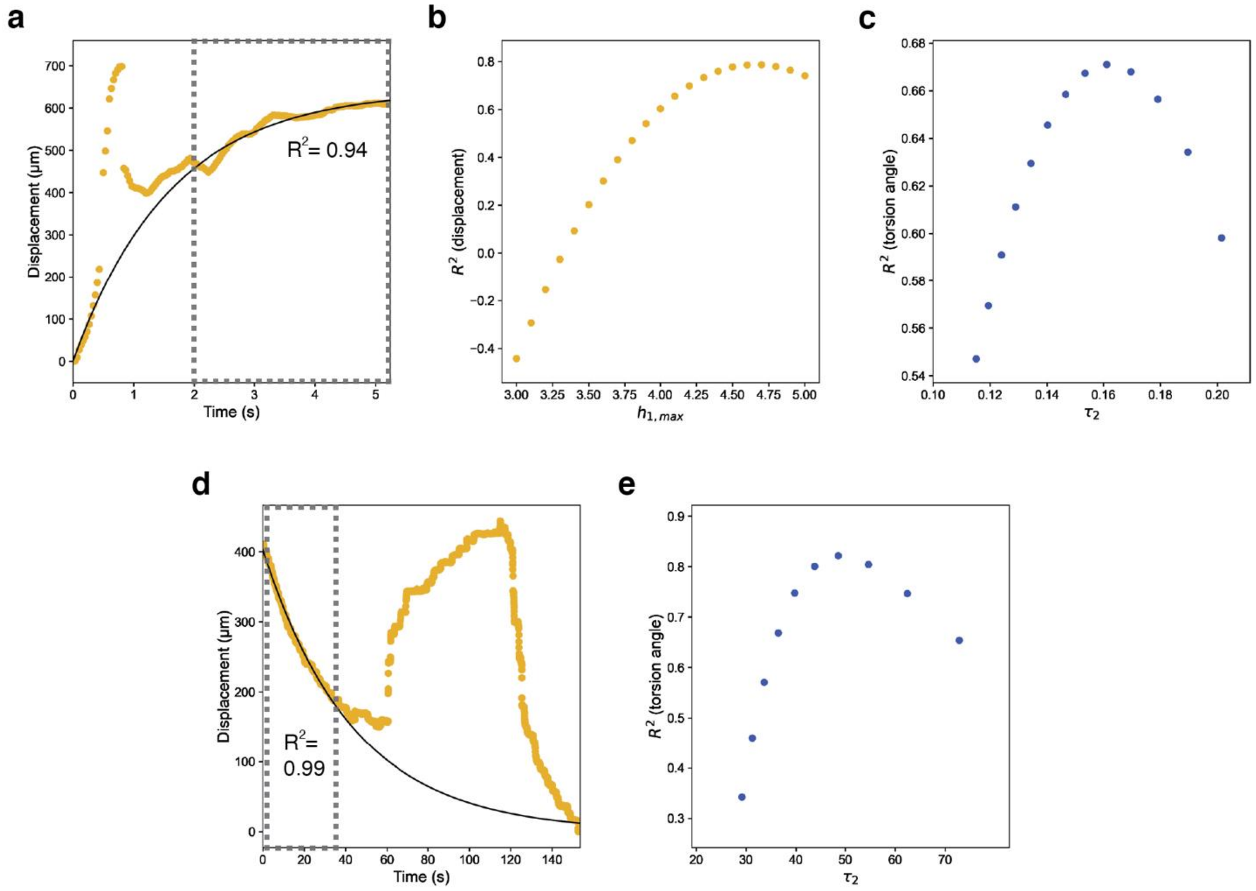

Supplementary Figure 12. Parameter optimization of FEA simulation when (001) face was irradiated. Optimization in the photo-process (**a**–**c**) and relaxation process (**d**–**e**). **a,d** Optimization of τ_{1p} (**a**) and τ_{1r} (**d**) based on R^2, where fitting to displacement was performed in the dotted area (optimized as τ_{1p} = 1.6 s and τ_{1r} = 43.7 s, respectively). **b** Optimization of $h_{1,max}$ based on R^2 to displacement (optimized as $h_{1,max}$ = 4.7 nm). **c,e** Optimization of τ_{2p} (**c**) and τ_{2r} (**e**) based on R^2 of torsion angle (optimized as τ_{2p} = 0.16 s and τ_{2r} = 43.7 s, respectively). Delay time in the photo-process and relaxation process was optimized manually as 0.39 s and 55 s, respectively.

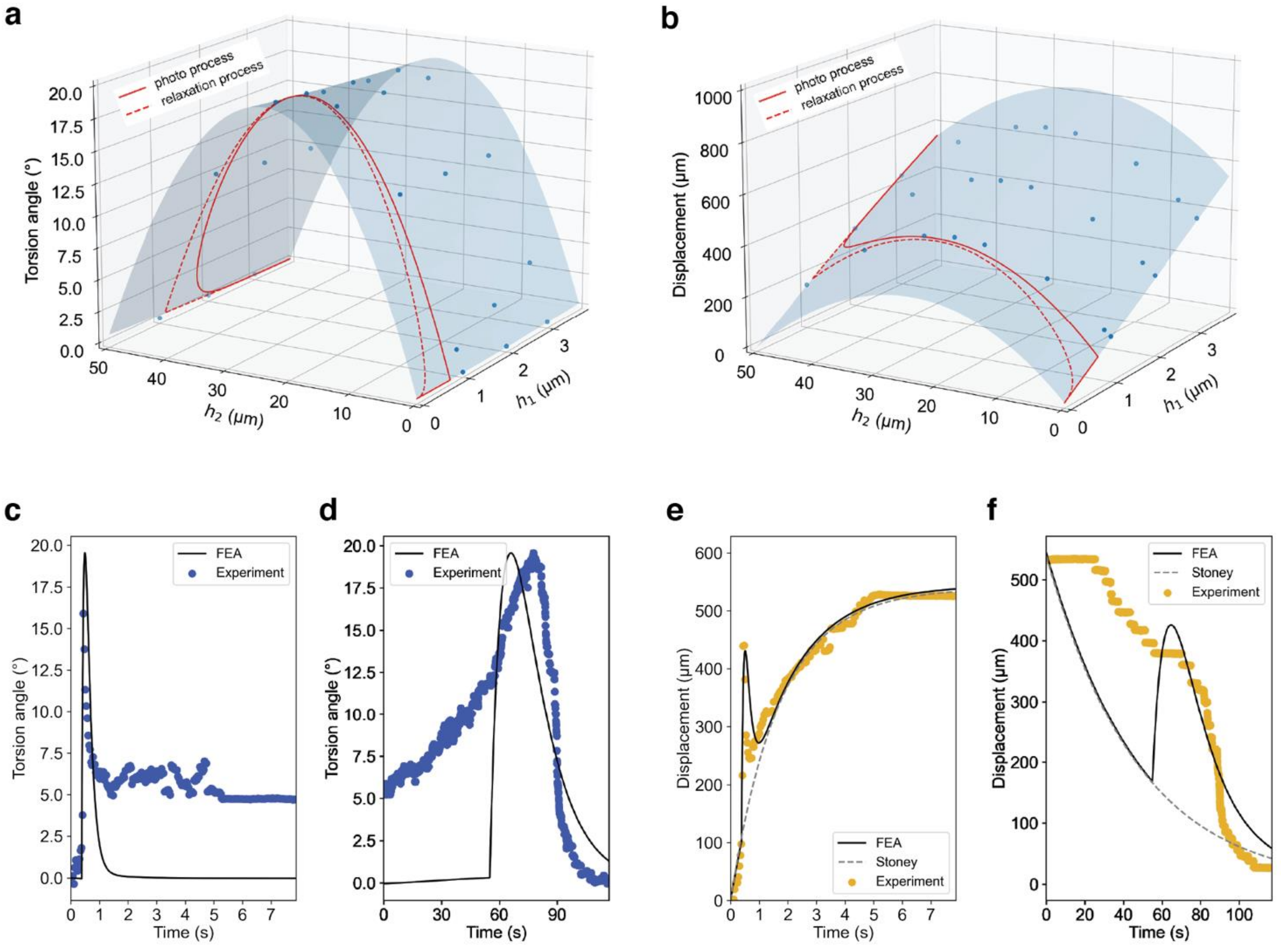

Supplementary Figure 13. FEA simulation of crystal deformation irradiated on (00$\overline{1}$) face. a,b Simulated dependance of torsion angle (**a**) and maximum displacement (**b**) on the thicknesses of h_1 and h_2 layers. Blue dots are the simulated data points, and the response surface are drawn by a polynomial function fitted to the simulated points. Red lines are the estimated route which reproduces the observed torsion angle and displacement. **c,d** Comparison of the simulation and the torsion angle in the photo-process (**c**) and relaxation process (**d**). **e,f** Comparison of the simulation and the displacement in the photo-process (**e**) and relaxation process (**f**). The experimental results come from Fig. 3h in the main text, and time was rescaled for each process.

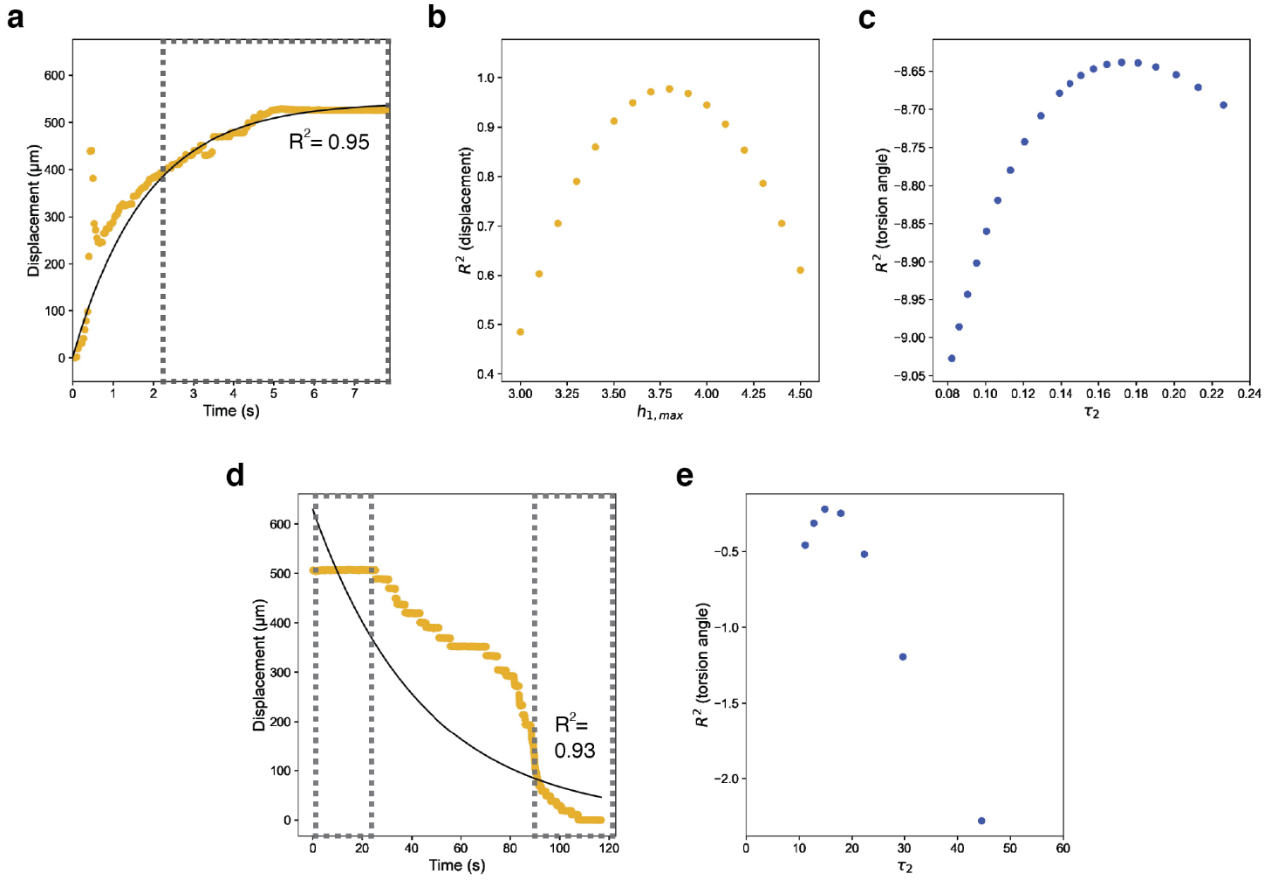

Supplementary Figure 14. Parameter optimization of FEA simulation when $(00\overline{1})$ face was irradiated. Optimization in the photo-process (**a–c**) and relaxation process (**d–e**). **a,d** Optimization of τ_{1p} (**a**) and τ_{1r} (**d**) based on R^2, where fitting to displacement was performed in the dotted area (optimized as τ_{1p} = 1.8 s and τ_{1r} = 44.6 s, respectively). **b** Optimization of $h_{1,max}$ based on R^2 to displacement (optimized as $h_{1,max}$ = 3.8 nm). **c,e** Optimization of τ_{2p} (**c**) and τ_{2r} (**e**) based on R^2 of torsion angle (optimized as τ_{2p} = 0.17 s and τ_{2r} = 14.9 s, respectively). Delay time in the photo-process and relaxation process was optimized manually as 0.38 s and 55 s, respectively.

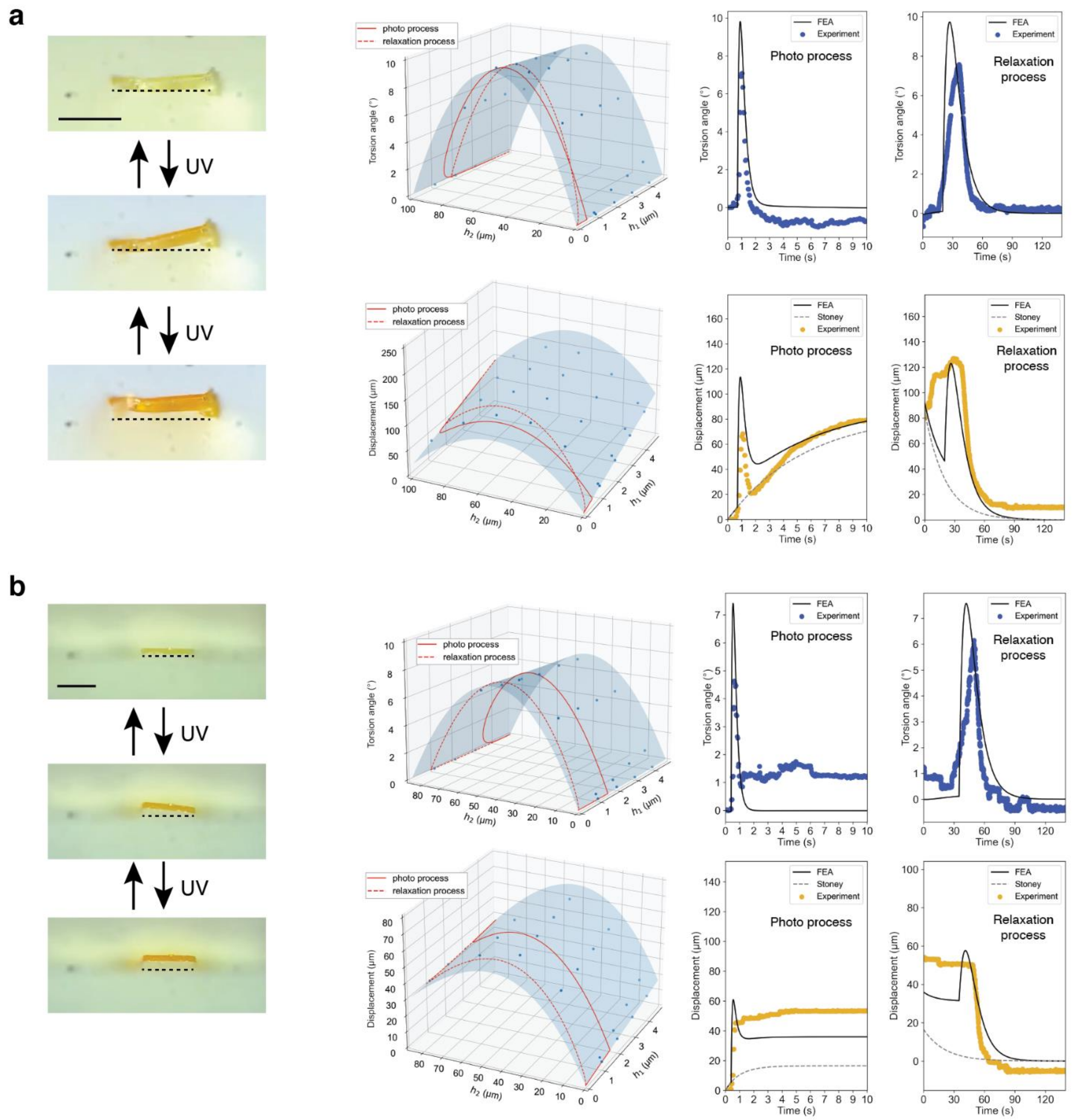

Supplementary Figure 15. Comparison of experiment and FEA-based simulation. a,b Cross-section view of deformation, simulated response surface of torsion angle and displacement, and comparison of observation and simulation. **a** Result of an enol-(*S*)-**1** crystal (2.79 mm, 0.79 mm, 100 μm). **b** Result of another crystal (1.06 mm, 0.69 mm, 86 μm). All scale bars are 500 μm.

4. Polarized microscopy

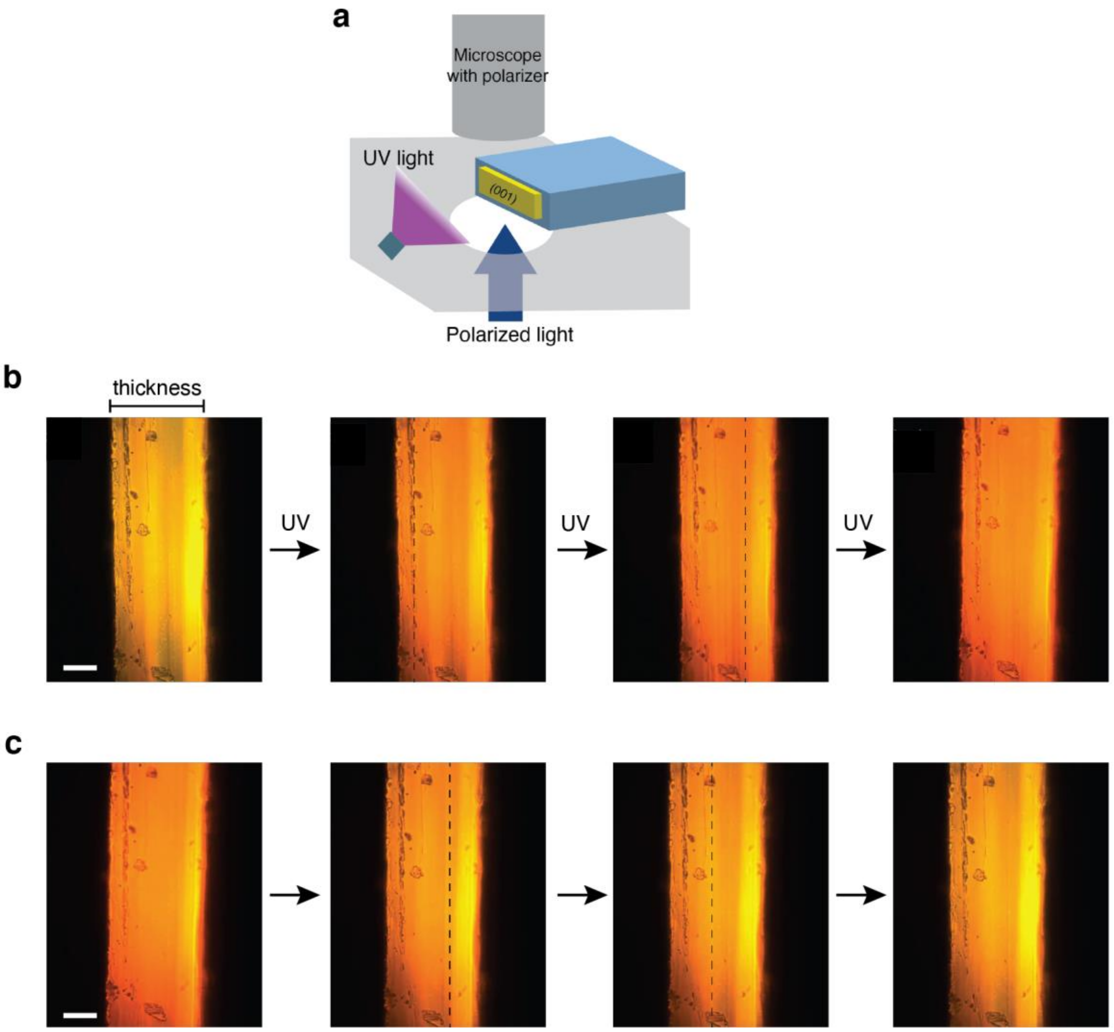

Supplementary Figure 16. Phase boundary at PtPT and reverse transition. a Schematic setup of the observation using polarized microscopy. **b,c** Progression and return of phase boundary parallel to (001) face upon light irradiation (**b**) and after the cessation (**c**). Dotted lines indicate phase boundary. Scale bars are 25 μm. Due to the small of change of birefrengence, phase boundary is more clearly seen in Supplementary Movie 5.

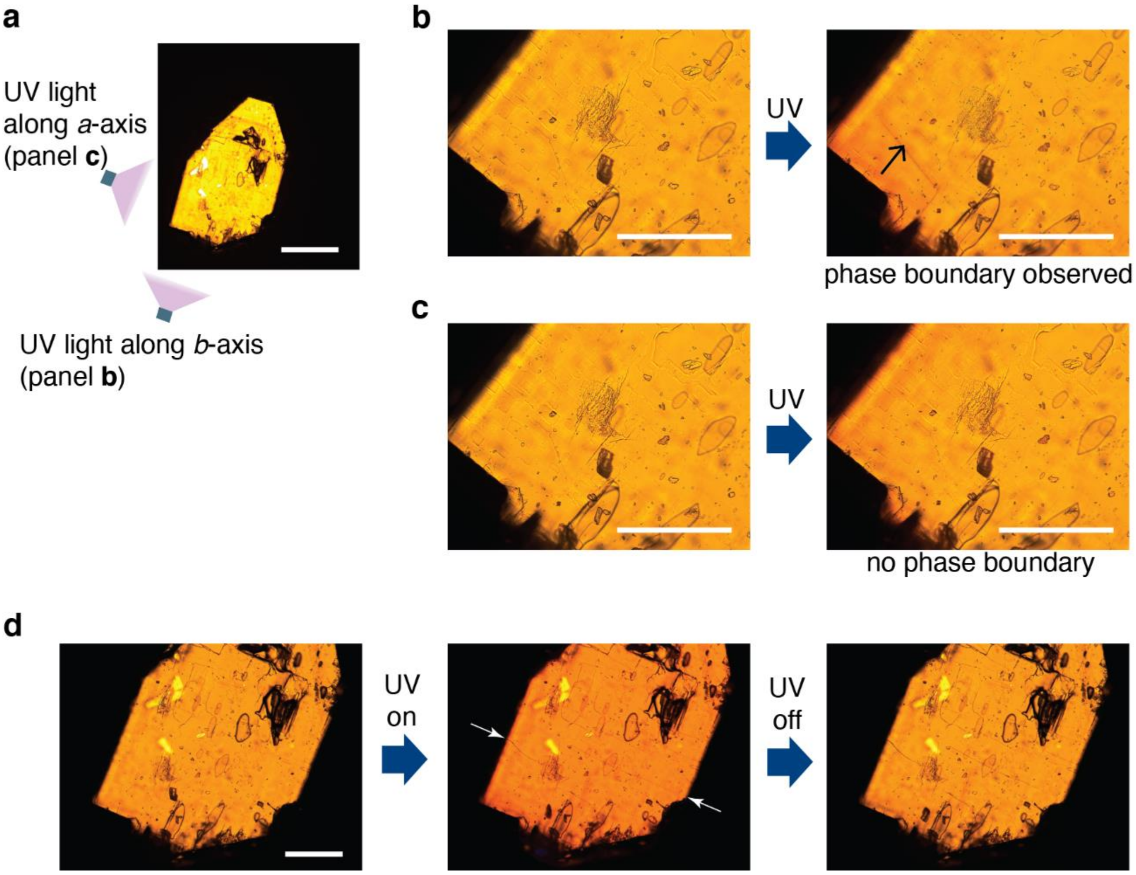

Supplementary Figure 17. Anisotropy of PtPT propagation. a (001) face view of an enol-(*S*)-**1** crystal under the polarized microscope. Scale bar is 500 μm. **b,c** Photographs of the crystal before and under UV light irradiation along *b*-axis (**b**) and *a*-axis (**c**). Scale bars are 100 μm. Light irradiation of 180 mW cm^{-2} was conducted parallel to the (001) face to avoid absorption from the (001) top surface. **d** Confirmation if local melting occurs by UV irradiation to (001) face at the highest intensity (360 mW cm^{-2}). Local melting did not occur while sruface cracks was generated as indicated by arrow. Scale bar is 250 μm.

5. Diffracted X-ray blinking (DXB)

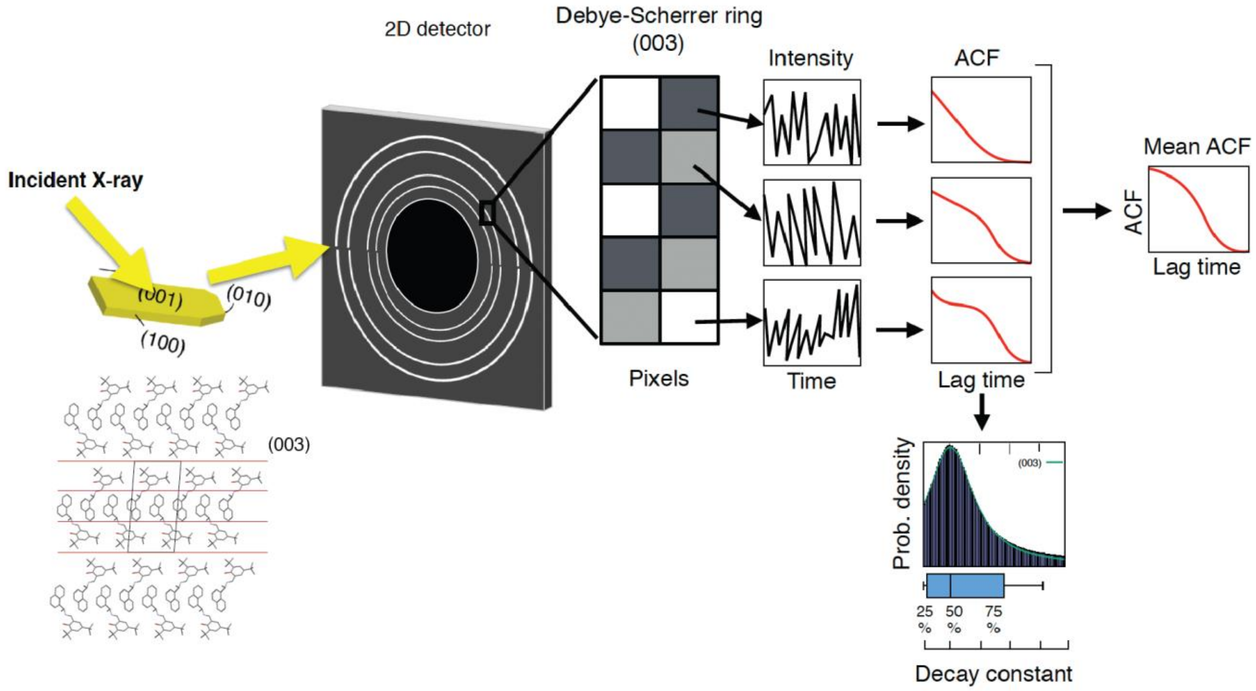

Supplementary Figure 18. Outline of DXB measurement. X-ray diffractions from enol-(*S*)-**1** powder were detected on 2D detector. Time-resolved intensity of each pixel on Debye-Scherrer ring of (003) plane was analyzed by autocorrelation function (ACF) with the variable of lag time. Based on the results of all pixels, mean ACF and probability density of decay constant can be obtained.

Supplementary Note 3. Explanation of DXB.

The outline of DXB measurement is shown in Supplementary Figure 18, and the results are shown in Figure 6 in the main text. In this Supplementary Note, more detailed explanation is mentioned. At first, in DXB measurement, the time-resolved images of X-ray diffractions are recorded. The time resolution, which depends on a used apparatus, is 50 ms in this research. When crystalline powder is the sample, we obtain many images of Debye-Scherrer rings by diffraction of the sample at the time resolution. All images are drawn by gray scale, where the stronger the intensity of X-ray diffraction, the closer to white.

When we focus on a specific Debye-Scherrer ring, (003) plane in this paper, we obtain diffraction intensities at all pixels along the circumference of the diffraction from the (003) plane. This diffraction ring was selected due to no overlap with other diffractions. In an image, the number of pixels is approximately 1000 pixels along (003) diffraction ring. The intensity value of a pixel, at a certain position, changes with time due to dynamics of crystal structure and noise. The intensity change should have hidden periodicity. Because the noise should arise randomly, periodicity of intensity value should be correlated with the dynamics of crystal structure. In the present case, it should be the dynamics of (003) plane like lattice fluctuation.

The hidden periodicity of intensity change at a certain pixel can be evaluated by autocorrelation function (ACF). As formulated by the equation (5) in the main text, ACF is the function of lag time. The ACF curve is obtained by changing the lag time, and then is fitted to an exponential curve, affording a decay constant. Thus, a decay constant is obtained by time-series intensity values at a certain pixel. Now, we have nearly 1000 pixels along (003) diffraction ring, and obtain corresponding decay constants from these pixels. The distribution of decay constants is visualized by histogram. The histogram shows raw information of the distribution but is difficult to be interpreted. For the interpretation, box plot is used and shows some statistics of the distribution, *ex.* median value. Based on these statistics, we can discuss the dynamics of crystal structure. Larger value of decay constant should indicate faster lattice fluctuation of the specific plane, and smaller value should correspond to slower dynamics.

In this research, we compared the distribution of decay constants in three states: before UV irradiation, initial few seconds upon UV irradiation, and long time under UV irradiation. As shown in Figure 6 in the main text, larger decay constants decreased during the initial few seconds upon UV irradiation. This change should correspond to the suppression of faster lattice fluctuation probably due to the stress induced by phase boundary, which is generated parallel to (003) plane.
