# Supplementary material for: Superelasticity of a photo-actuating chiral salicylideneamine crystal
Source: Commun Chem. 2022 Jan 10;5:4. doi: 10.1038/s42004-021-00618-8 (PMC9814393; doi:10.1038/s42004-021-00618-8)
Supplement: Supplementary file 8 — Description of Additional Supplementary Files [file 42004_2021_618_MOESM8_ESM.pdf]

Description of Additional Supplementary Files

File name: Supplementary Movie 1
Description: Cross-section view of stepwise twisted actuation of a crystal whose (001) face was irradiated by UV light.

File name: Supplementary Movie 2
Description: Cross-section view of stepwise twisted actuation of a crystal whose $(00\bar{1})$ face was irradiated by UV light.

File name: Supplementary Movie 3
Description: Free deformation and blocking force measurement upon UV light irradiation on $(00\bar{1})$ face.

File name: Supplementary Movie 4
Description: Free deformation and blocking force measurement upon UV light irradiation on (001) face.

File name: Supplementary Movie 5
Description: Polarized microscopy of a crystal glued to a glass substrate for the prevention of free deformation, upon UV light and after the cessation.
